# Supplementary material for: Targeted delivery of CRISPR-Cas9 ribonucleoprotein into arthropod ovaries for heritable germline gene editing
Source: Nat Commun. 2018 Aug 1;9:3008. doi: 10.1038/s41467-018-05425-9 (PMC6070532; doi:10.1038/s41467-018-05425-9)
Supplement: Supplementary file 1 — Supplementary Information [file 41467_2018_5425_MOESM1_ESM.pdf]

Chaverra-Rodriguez et al. 2018. Targeted delivery of CRISPR-Cas9 ribonucleoprotein into arthropod ovaries for heritable germline gene editing

Supplementary Figures

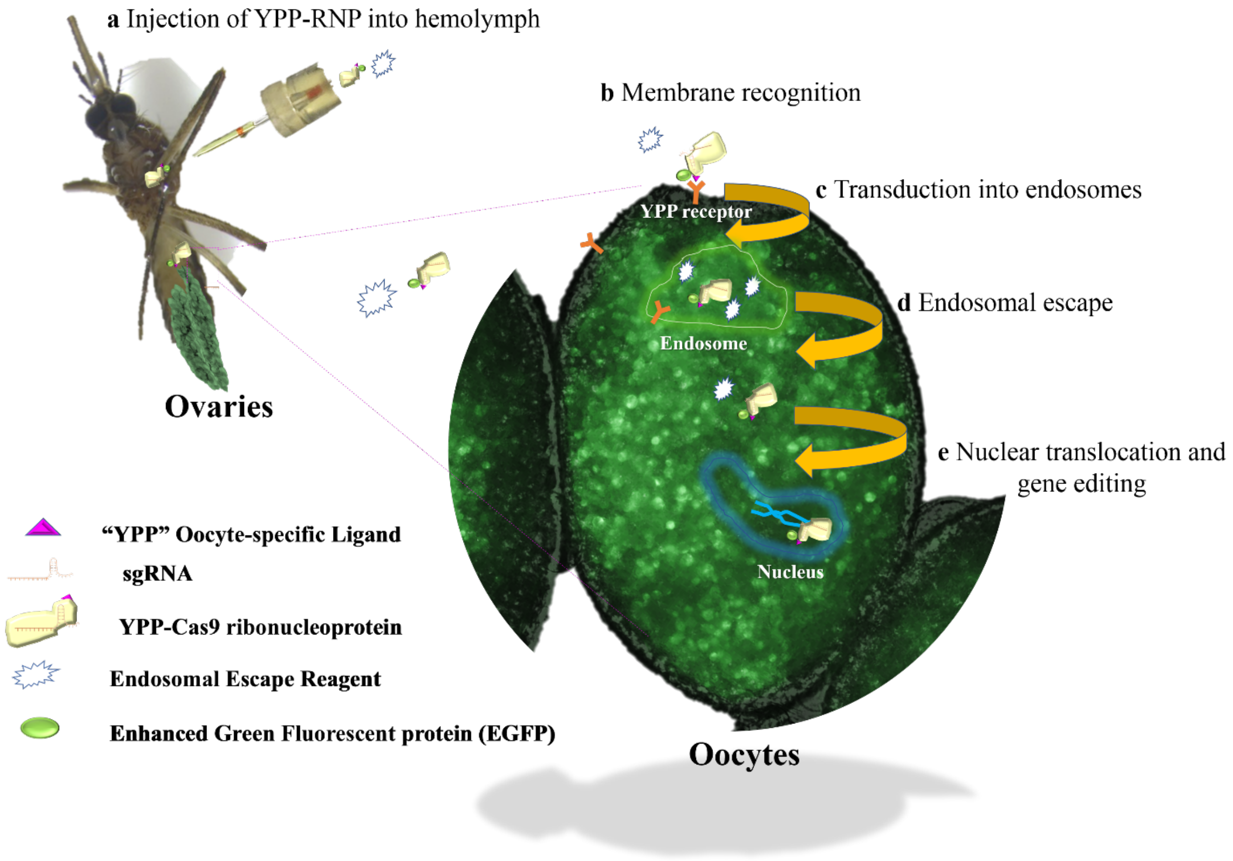

Supplementary Figure 1. Rationale for Receptor-Mediated Ovary Transduction of Cargo (ReMOT Control). After a female mosquito takes a blood meal, yolk protein receptors are expressed on the membrane of the developing oocytes in the ovaries. **a** A capillary needle is used to inject into the female's hemolymph a solution containing a Yolk Protein Precursor (YPP) fused to Cas9 ribonucleoprotein (YPP-Cas9 RNP) mixed with an endosomal escape reagent (EER). **b** The YPP ligand domain of the fusion protein is recognized by receptors in the oocyte membrane. **c** The YPP-Cas9 is internalized into endosomes where it is sorted to yolk granules and lysosomes. **d** The addition of membrane destabilizing reagents allow YPP-Cas9 to escape the endosome and reach the cytoplasm, the nuclear localization signal present in Cas9 directs the enzyme to the nucleus. **e** The molecules that reach the nucleus bind the DNA and create double strand breaks in the oocyte's chromosome. EGFP may be fused to the YPP-RNP for tracking purposes.

a

pAc5-DmYPss-EGFP

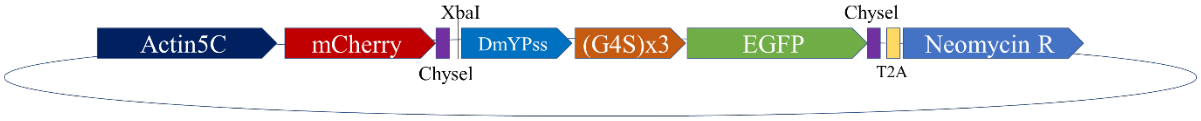

DmYPss amino acid sequence

MNPMRVLSLLACLAVAALAKPNGRMDNSVNQALKPSQWLSGSQLEAIPALDDFTIERLENMNLERGAELL

b

pAc5-DmYPss-DmYP1-EGFP

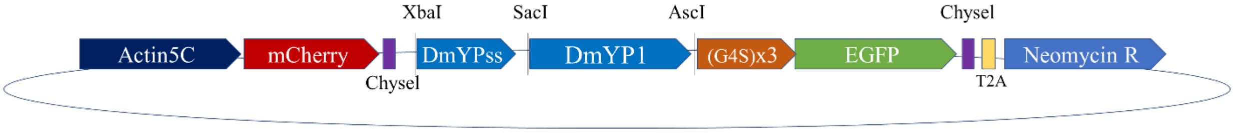

DmYP1 amino acid sequence

HLSQIHHNVEPNYVPSGIQVYVPKPNGDKTVAPLNEMIQRLKQKQNFGEDEVTIIVTGLPQTSETVKKATRKLVQAY
MQRY*NLQQQRQHGKNGNQDYQDQSNEQRKNQRTSSEEDYSEEVKN*AKTQSGDIIVIDLGSKLNTYERYAMLDIEKTG
AKIGKWIVQMVNELDMPFDTIHLIGQNVGAHVAGAAAQEFTRLTGHKLRRVTGLDPSKIVAKSKNTLTGLARGDAE
FVDAIHTSVYGMGTPIRSGDVDFYPNGPAAGVPGASNVVEAAMRATRYFAESVRPGNERSFPAVPANSLQQYKQND
GFGKRAYMGIDTAHDLEGDYILQVNPKSPFGRNAPAQKQSSYHGVHQAWNTNQDSKDYQ

c **d**

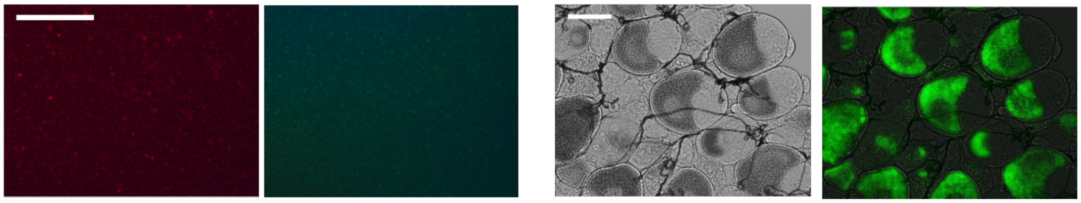

Supplementary Figure 2. Plasmids maps and protein expression in *D. melanogaster* S2 cells. **a** Plasmid map of pAc5-DmYPss-EGFP. The amino acid sequence for DmYPss is showed below the map, the first 20 amino acids (in blue) represent the signal peptide used for secretion of any downstream protein. **b** Plasmid map of pAc5-DmYPss-DmYP1-EGFP. The amino acid sequence for the protein is showed below the plasmid map highlighting in different colors three portions that were further tested for EGFP transduction. Green is portion 2 (P2, italicized text represents the sequence for P2C), magenta is portion 3 (P3) and black is portion 4 (P4). The whole sequence for DmYP1 includes DmYPss+P2+P3+P4. **c** Expression of mCherry (left) and DmYP1-EGFP (right) in S2 cells transfected with the plasmid pAc5-DmYPss-DmYP1-EGFP. Scale bar = 50 µm. **d** Transduction of S2-expressed DmYP1-EGFP into the oocytes of *An. gambiae* (N = 20, also see supplementary Table 1). Scale bar = 100µm.

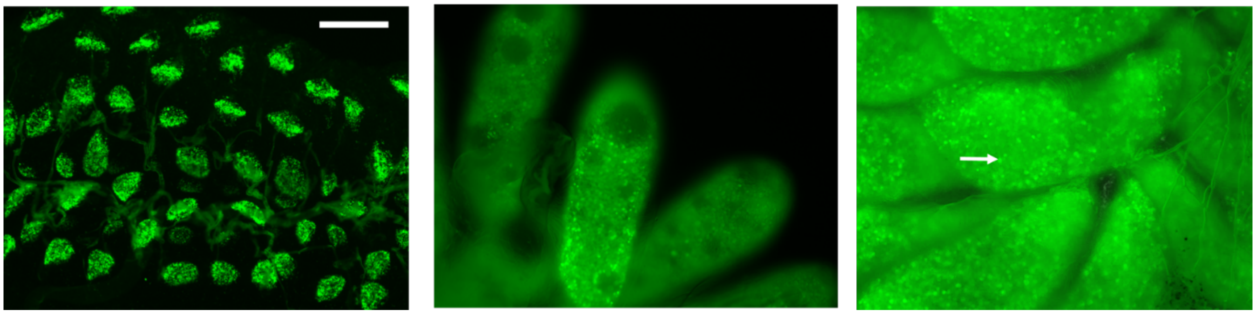

Supplementary Figure 3. Characterization of DmYP1-mediated transduction of EGFP. Transduced EGFP is observed in yolk granules throughout oocyte development. Left. 24h PBF. Center 36h PBF. Right 48h PBF (white arrow indicates yolk granules). Scale bar = 100 μm.

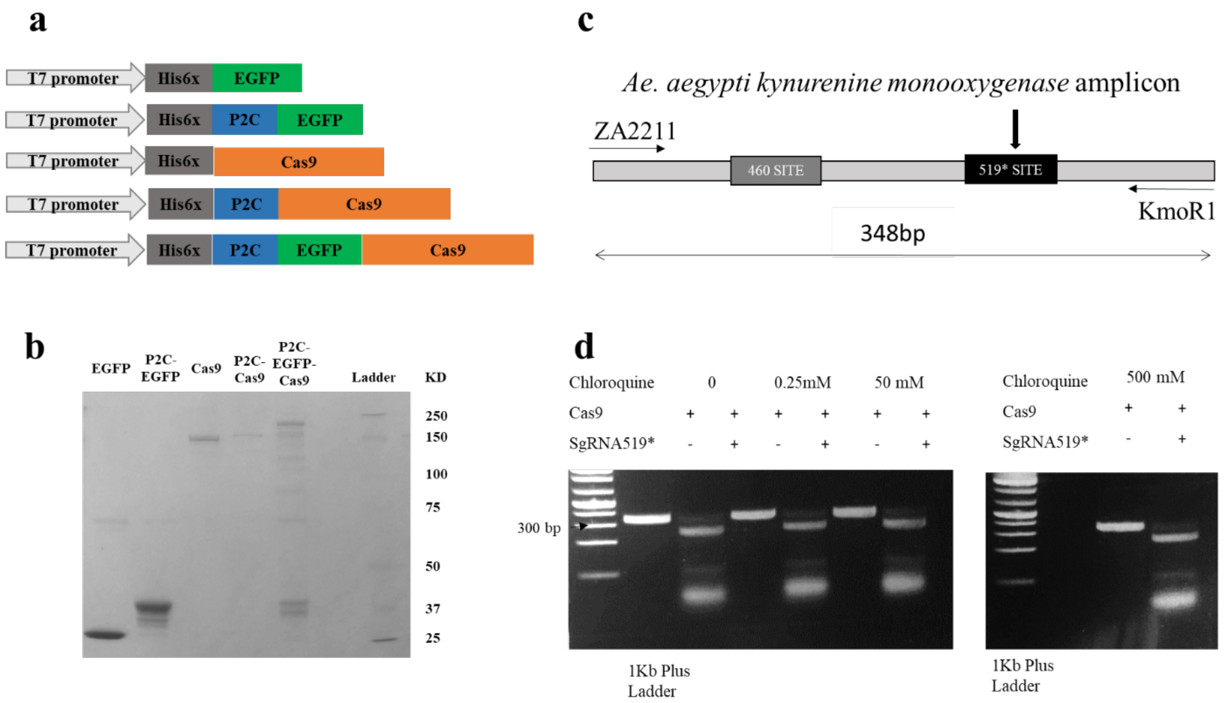

Supplementary Figure 4. Verification of *E. coli* expression and activity for recombinant proteins used in this study. **a** Construct maps for different proteins. **b** Coomassie-stained gel of *E. coli*-expressed EGFP, P2C-EGFP, Cas9 and P2C-EGFP-Cas9. **c** Schematic showing cut site for a 348bp amplicon of the *kmo* gene used for *in vitro* Cas9 cleavage assays. **d** Agarose gel (1%) showing the DNA fragments resulted from the *kmo* amplicon cleavage assay using P2C-EGFP-Cas9 and sgRNA519* mixed with chloroquine. In the absence of sgRNA the amplicon is not cleaved. (+) indicates that the component is present (–) indicates that the component was not added to the mix.

Supplementary tables

Supplementary Table 1. Effects of time of injection on delivery efficiency.

Time of injection (hours PBF)/ Replicate* #	♀ Injected (N)	♀ showing EGFP in follicles (N)	♀ showing EGFP in follicles (%)	Follicles observed** (N)	Follicles with EGFP (N)	% Follicles with EGFP (Efficiency)***
12h/1	20	17	85%	243	243	100%
12h/2	20	19	95%	383	381	98%
Total	40	36	90%	626	624	99.7%
24h/1	20	18	90%	250	245	98%
24h/2	20	17	85%	200	197	99%
Total	40	35	88%	450	442	98.2%

*The experiment was replicated twice for each time of injection. No statistical analysis was performed on this data.

**A subsample of females (N = 5-8) showing EGFP in the ovaries were dissected, 50 follicles were randomly chosen and scored for EGFP detection under microscopy.

***Efficiency was similar in further trials, we noticed that a female that showed EGFP in its ovaries presented high rates of follicle transduction.

Supplementary Table 2. Validation of *in vivo* activity for fusion P2C-Cas9/sgRNA460 RNPs by standard embryo injection.

Protein	[Protein] (ng μL^{-1})	[sgRNA] (ng μL^{-1})	Eggs injected (N)	G_0 Larvae hatched (N)	% hatching	G_0 Mosaic (N)	G_0 white (N)	#KO/ injection EEF (%)	#KO/ Surviving G_0 GEF (%)
Cas9	300	100	48	23	48%	1	0	2.1%	4%
			48	21	44%	1	0	2.1%	5%
			73	23	32%	7	0	9.6%	30%
		Total	169	67	39.64%	9	0	5%	13%
Cas9-P2C	300	100	101	34	34%	0	0	0	0
			74	21	28%	0	0	0	0
	300	200	224	47	21%	19	2	9.4%	45%
		Total	399	102	25.5%	19	2	5%	21%
Cas9-P2C-EGFP	300	250	137	53	39%	0	0	0	0
	300	200	100	13	13%	1	1	2.0%	15%
		Total	237	662	28%	1	1	1%	3%

Supplementary Table 3. Optimization of the injection conditions required for gene editing of the maternal chromosome with P2C-EGFP-Cas9/sgRNA519* RNP without EERs.

[Protein] ng μL^{-1}	[sgRNA] ng μL^{-1}	Time (h)	EER	N	N Surviving	N laying*	N eggs	N larvae	N Females laying KO**	N Knockouts
2700	1750	24	**NONE**	20	11	1	30	2	0	0
1867	1853	24	**NONE**	12	10	7	235	231	0	0
1867	1853	24	**NONE**	17	9	3	68	18	0	0
1867	1853	24	**NONE**	17	15	9	454	210	0	0
1867	1853	48	**NONE**	10	6	3	128	73	0	0
3337	1167	30	**NONE**	9	7	<u>6</u>	166	75	0	0

*After 24h of injection, surviving females were allowed to lay eggs individually (underlined numbers) or in a single group of survivors (no text formatting). The number of females laying eggs was determined directly from isofemales, or estimated as the number of females alive when eggs first appeared in the group cages.

**The number of females laying knockouts (KO) was 1 when only one KO was produced from group of females or was determined directly from isofemales

Supplementary Table 4. Optimization of injection conditions required for gene editing of the maternal chromosome with P2C-EGFP-Cas9/sgRNA519 RNP and Chloroquine.

[RNP] ng µL^{-1}	[sgRNA] ng µL^{-1}	Time (h)	EER	N	N Surviving	N laying*	N eggs	N larvae	N Females laying KO**	N Mosaic	N White
3337	1167	30	Chlq-0.25mM	15	8	6	126	72	0	0	0
3337	1167	30	Chlq-0.5mM	15	10	6	123	79	1	0	1
3337	1167	30	Chlq-1mM	15	9	5	63	46	0	0	0
3882	1342	28	Chlq-0.5mM	39	17	17	633	375	2	0	2
3375	**703**	28	Chlq-0.5mM	30	25	17	405	137	0	0	0
3375	**703**	28	Chlq-0.5mM	25	20	20	573	330	0	0	0
3375	**703**	28	Chlq-1mM	30	25	20	567	365	0	0	0
3375	**703**	28	Chlq-10mM	30	27	23	532	351	0	0	0
3375	**703**	28	Chlq-0.5mM	20	20	18	497	265	0	0	0
3375	1353	54	Chlq-0.5mM	36	34	30	ND	567	0	0	0
3375	1353	54	Chlq-1mM	36	29	29	ND	579	1	1	0
3375	1353	54	**Chlq-20mM**	28	17	17	ND	220	0	0	0
2121	1098	54	Chlq-0.5mM	32	28	28	720	149	1	1	0
2121	1098	54	Chlq-1mM	31	25	25	314	162	1	0	1
2121	1098	54	Chlq-2mM	22	20	20	412	228	1	0	1
6750	1406	54	Chlq-0.5mM	13	11	11	300	143	1	0	1
3375	1353	24	Chlq-0.5mM	36	19	11	262	87	1	0	3
3587	1737	54	**Chlq-20mM**	20	19	19	500	440	0	0	0
4214	**903**	24	Chlq-0.5mM	62	28	12	459	105	0	0	0
3375	1353	28	Chlq-0.5mM	15	12	8	418	330	0	0	0
3375	1393	**5**	Chlq-0.5mM	19	18	13	766	676	0	0	0
3375	1393	**5**	Chlq-0.5mM	20	19	10	666	574	0	0	0
3375	1393	24	Chlq-0.5mM	15	12	10	500	379	0	0	0
**1588**	1405	24	Chlq-0.5mM	26	24	18	994	816	0	0	0
**1125**	1393	24	Chlq-0.5mM	26	26	19	982	809	0	0	0
**502**	1393	24	Chlq-0.5mM	26	24	13	602	457	0	0	0

*After 24h of injection, surviving females were allowed to lay eggs individually (underlined numbers) or in a single group of survivors (no text formatting). The number of females laying eggs was determined directly from isofemales, or estimated as the number of females alive when eggs first appeared in the group cages.

**The number of females laying knockouts (KO) was 1 when only one KO was produced from group of females or was determined directly from isofemales

Bold text indicates conditions tested that did not work at least once and thus were not further tested.

Supplementary Table 5. Life history parameters for isofemales injected with Cas9, P2C-Cas9 and P2C-EGFP-Cas9.

RNP	[RNP] ng μL^{-1}	N injected	Survival	% Survival	♀ Laying*	% Laying	Eggs	G_0WT	G_0 KO
Cas9	2250	25	18	72%	19	76%	835	459	0
	2250	30	30	100%	28	93%	1339	868	1
	2250	28	21	75%	13	46%	nd	200	0
	2250	25	20	80%	22	88%	1179	675	0
	2250	30	30	100%	19	63%	758	415	0
	Total	138	119	86%	101	73%	4111	2617	1
P2C-Cas9	2200	25	17	68%	17	68%	972	645	7
	2200	30	30	100%	23	77%	1084	665	0
	2200	25	19	76%	18	72%	994	531	21
	2200	29	29	100%	21	72%	937	521	4
	Total	109	95	87%	79	72%	3987	2362	32
P2C-EGFP-Cas9	2375	27	27	100%	24	89%	1144	795	0
	2375	15	15	100%	14	93%	638	409	1
	Total	42	42	100%	38	90%	1782	1204	1

Data on each row for the same RNP represents a replicate.

*Note: some females that appeared to be dead 24 hours PI were not dead and laid eggs.

Supplementary Table 6. Gene editing of the maternal allele using several Cas9 RNP at different concentrations and endosomal escape reagents (EER). One trial consisted of 10-40 females.

Protein	[Protein] ng μL^{-1}	[sgRNA] ng μL^{-1}	Endosomal Escape Reagent	N Trials	♀ Injected	G_0 WT	G_0 KO	#KO/ injection EEF (%)	#KO/ Surviving G_0 GEF (%)
Cas9	1670	639	None	1	10	42	0	0.0%	0.00%
	1466-2952	1062-1253	Chloroquine 0.5-5mM	5	138	2617	1	0.7%	0.04%
			Total	6	148	2649	1	0.7%	0.04%
P2C-Cas9	2250	1400	Chloroquine 0.5 mM	5	142	2850	43	30%	1.51%
P2C-EGFP-CAS9	1237-3337	1118-1853	None/dynasore	6	117	599	0	0.0%	0.00%
	2258-6750	703-1406	Chloroquine 0.25-10mM	25	672	8156	12	1.8%	0.15%
	3375	703	Monensin 1mM	1	20	405	0	0.0%	0.00%
	3375	703	Saponin 24μM	1	20	164	0	0.0%	0.00%
	3375	703	NH_4Cl 50mM	1	20	265	0	0.0%	0.00%

Supplementary Table 7. Gene editing of the paternal allele using P2C-EGFP-Cas9 RNP at different concentrations and endosomal escape reagents (EER). One trial consisted of 10-40 females.

[Protein] ng μL^{-1}	[sgRNA] ng μL^{-1}	Endosomal escape strategy	N Trials	♀ injected	Wild type	White	#KO/ injection EEF (%)	#KO/ Surviving G_0 GEF (%)
1250	1863	None	5	73	266	1	1%	0%
3000-3375	1040-1167	Chloroquine 0.5-1mM	4	80	464	0	0%	0%
3375	1040	Monensin 1mM	1	20	53	1	5%	2%
3375	1040	Saponin 24µM	1	20	160	1	5%	1%
3375	1040	NH_4Cl 50mM	1	20	29	0	0%	0%

Supplementary Table 8. Heritability test of the mutations in the *kmo* site 519. Individual G_0 adults showing a knockout phenotype were mated to white eye adults.

# Test	G_0 adults				G_1 Offspring (N)*		
	ID	Phenotype	Sex	# Egg batches	Wild type	White	% White eye
**1**	Mon-KO8	Mosaic	Male	1	18	17	48.57%
**2**	Sap-KO11	Mosaic	Male	1	14	16	53.33%
**3**	KO-2021-1.8	White	Female	1	0	61	100%
				2	0	52	100%
**4**	KO6-2	White	Male	1	0	51	100%
**5**	KO6-3	White	Female	1	0	23	100%

*If the mutations are heritable, these will appear in >75% of G_1 larvae. White eye G_0, but not mosaics, showed 100% white eye progeny.

Supplementary Methods

Plasmid construction and protein expression in S2 cells

D. melanogaster cell line S2 (ATCC® CRL-1963™) was used for protein production. The plasmid pAc5-STABLE1-neo[91] was previously modified in our lab to introduce the exon 1 of DmYP1 containing a secretion peptide signal (DmYPss) creating the vector pAc5-DmYPss-EGFP and also the plasmid pAc5-DmYPss-DmYP1-EGFP (Supplementary Figure 2, sequences of the inserts are available in the Supplementary Notes). For protein expression, the plasmids pAc5-DmYPss-(G4Sx3)-EGFP and pAc5-DmYPss-DmYP1-EGFP was transiently transfected into S2 cells, on 6 well plates, using 2 ml of Schneider's medium (Thermofisher) containing 3-5x10^6 cells per well. Briefly, 2.5 µg of plasmid DNA were diluted into 500 µL of Opti-MEM® I Reduced Serum Medium without serum, added 2.5 µL of Plus Reagent to diluted DNA and mixed gently, incubated 5 – 15 min at room temperature. 5 µL of Lipofectamine® with Plus™ LTX (ThermoFisher) was added into the above-diluted DNA solution, mixed gently and incubated for 25 minutes at room temperature. 500 µL of the DNA-lipofectamine complexes were added directly to each well containing cells and agitated gently by rocking the plate back and forth. Cells were incubated at 25°C for 24–72 h. The production of EGFP alone was used as control for secretion (plasmid pAc5-EGFP). Transfection rates were assessed by scoring the percentage of cells expressing mCherry under fluorescence microscopy. The secreted fusion DmYP1-EGFP protein was collected from medium 72 h post-transfection by spinning down the cell culture at 1000g x 3 min and concentrating the supernatant 100 times (100X) using Centricon plus-70 filter units of 30kD. The concentrated supernatant was used directly for injection or stored at -80C. Additionally, for stable transfection, 10% fetal Bovine serum (FBS) and 2000 µg/ml Geneticin® G418 sulfate (Thermofisher) were added to the initial medium for transfection, and weekly for selection of transformed cells, once stable, the cells were maintained without G418.

Identification of a putative receptor-binding region of DmYP1 for *An. gambiae* ovaries. A deletion analysis of the DmYP1 protein was conducted to identify the smaller region that efficiently transduced cargo into *An. gambiae* ovaries. Different fragments of the protein DmYP1 (Figure 1a) were amplified with primers containing the restriction site SacI and fused to EGFP using the backbone plasmid pAc5-DmYPss-EGFP. The fusion protein containing the fragment of 439 amino acids (DmYP1) was used as a positive control, recombinant EGFP without a targeting ligand was used as a negative control. Five portions of the gene were tested to find the RBR, First, the gene was divided in three equal portions each of approximately 120 amino acids termed P2, P3 and P4, numbered from the NH-terminus to the COOH-terminus respectively. Portion five (P5) included P2 and P3 and portion six (P6) included P3 and P4 (Figure 1a).

All the procedures including cell transfection, protein expression and purification were conducted as described above. Groups of 10-20 females were injected with each protein fragment. Ovaries were dissected 12 and 48 hours post-injection and visualized for EGFP

fluorescence. 50-100 follicles were examined. The experiment was repeated two times. The efficiency of EGFP delivery was estimated as the percentage of oocytes showing EGFP in the yolk granules.

Optimization of conditions for maternal gene editing injections

RNP preparation

RNP was prepared using a molar ratio protein to sgRNA between 1:2-1:3. Variation in the concentrations of RNP and sgRNA, time of injection post-blood feeding and Endosomal escape reagents (EER) were tested. Final RNP concentration ranged between 500 ng μL^{-1} to 6000 ng μL^{-1}. sgRNA concentration ranged from 700 to 1853 ng μL^{-1}, time of injection between 5 to 54 hours PBF. Several EER were tested. Chloroquine was used at different concentrations between 0.25 to 20 mM. Chloroquine was prepared from a stock at 70 mM kept at -80 ºC. After several trails without positive results, we noticed that working solutions of the EER chloroquine were not appropriate for short-term storage. **These solutions should be prepared fresh for each trial, and added to the RNP mix immediately before injections.**

Selection of females for outcrossing and injection

For optimization, wild type (WT) females were outcrossed to white-eye (Wh-Iso8-kmo460) males. To obtain these adults, eggs from both colonies were hatched during the same day and were provided with food until pupation. Pupae were sorted by sex under a dissecting microscope and placed inside cages labeled with the mating type, number and sex of the pupae. The cages containing WT female pupae were observed daily to carefully monitor emergence and remove any male adult that could potentially affect the experiment. One-day old white eye adult males were transferred to the cage with WT females. Mating was allowed during at least 4 days. For injections, females 5-7 days old were fed in the cage. The day of injections females were placed at 4 ºC and sorted by feeding status, we also checked that there was no contamination with wild type males or white-eye females in each cage before proceeding with injections.

Survival and oviposition of injected females

After 24h of injection, surviving females were transferred to oviposition containers to lay eggs individually (isofemales) or in a single group of survivors (group). Survival was determined after examining the females after injections, those individuals that did not move, fly or respond to manipulation with forceps were considered dead. Procedures related to determining survival, fecundity and fertility of each treatment are described below.

Survival and oviposition in groups. All the females that survived the injections were placed inside a cage containing an oviposition container consisting of a plastic cup with a paper filter soaked in reverse osmosis (RO) water. Sucrose solution (10%) was provided in a cotton ball.

Survival was measured daily, and the day of first egg laying was recorded. On day three after the first egg was laid, surviving females were transferred to a new cage and the dead ones were transferred to microvials and frozen at -80 ºC. The paper filter containing eggs was removed and let dry for 12-24 h and the eggs were hatched on 500 ml of RO water supplemented with one or two grounded fish food pellets for the hatching larvae.

Survival and oviposition of isofemales. Females were placed on oviposition containers made with 50 ml Falcon® tubes (VWR). A cotton ball soaked on 2 ml of RO water was placed at the bottom of each tube with Whatman filter paper (VWR) placed on top of it. A nylon mesh wrapped with rubber bands was used to cover the tube and a cotton ball soaked on 10% sterile sugar solution was provided on top of each mesh. Survival and oviposition was measured daily after isolating the females. On day three after the first egg was laid, surviving females were transferred to a new oviposition tube and the dead ones were transferred to microvials and frozen at -80 ºC. The cotton ball and the paper filter containing eggs were removed, the eggs were dried for 12-24h, then the eggs were placed back in the tube and immersed in 10-15 ml of RO water supplemented with 100 µL of 1% ground fish food slurry prepared in RO water provided as food for the hatching larvae.

Number of females laying eggs. The number of females laying eggs, the number of eggs per female, the number of females laying mutants and the number of mutants per female were directly determined from isofemale experiments. In the group cages the number of females laying eggs was estimated as the number of females alive when eggs first appeared, the number of females laying knockouts (KO) was 1 only when 1 KO was produced from the group of females, otherwise it was not determined (ND). The number of mutants per female was not determined unless there was only one mutant from the group of females.

Screening

Larvae hatching was confirmed 24h-48h after soaking the eggs in water, those eggs that did not hatch were dried for 12h-24h and soaked in RO water. 48 hours after hatching was confirmed, larvae were placed on a home-made sifter made with a plastic petri dish which had the bottom removed and replaced with a fine nylon mesh that retained the larvae but not water. The progeny of each isofemale was independently screened for white eye or mosaic larvae, as well as the total progeny of the females that were grouped. The mutants were isolated in tubes labeled with the treatment, day and mutant ID and allowed to become adults. Once finished screening, the wild type larvae were returned to the tubes and allowed to grow to pupae to perform a second screening for white eye or mosaics that were not detected initially. Screening early larvae instead of waiting to screen at the pupal stage allowed us to detect mutants that could die before they reach that stage and thus allowing us to better estimate the gene editing efficiency of ReMOT Control.

Supplementary Note1: DNA sequences (5' to 3') for protein expression of fusion proteins DmYP1 and derivatives on S2 cells using the backbone vector pAc5-STABLE1-neo. Highlighted sequence in light blue is DmYP1 exon 1 that contains the secretion signal, grey is the G4Sx3 linker, dark red is DmYP1 or any of its derivatives (P2-to P6 and P2A, P2B or P2C), green is EGFP. Lowercase letters indicate restriction sites used for cloning the fragments or nucleotide additions required to make the protein in-frame.

pAc5-DmYPss-EGFP

tctagaATGAACCCCATGAGAGTGCTGAGCCTTCTGGCTTGCTTGGCGGTCGCCGCCTTG
GCCAAGCCCAATGGCCGTATGGACAACTCCGTCAACCAGGCATTGAAGCCGTCGCA
GTGGCTCTCCGGATCCCAGCTGGAGGCCATTCCCGCCCTCGACGATTTCACCATTGA
GCGTCTGGAGAACATGAACCTGGAGCGTGGCGCCGAGCTGCTGCAGCAAGTATACG
GGCCCgagctcAGGCGCGCCGGAGGAGGCGGCAGCGGTGGTGGAGGAAGCGGTGGAG
GCGGGAGCATGGTGAGCAAGGGCGAGGAGCTGTTCACCGGGGTGGTGCCCATCCTG
GTCGAGCTGGACGGCGACGTAAACGGCCACAAGTTCAGCGTGTCCGGCGAGGGCGA
GGGCGATGCCACCTACGGCAAGCTGACCCTGAAGTTCATCTGCACCACCGGCAAGC
TGCCCGTGCCCTGGCCCACCCTCGTGACCACCCTGACCTACGGCGTGCAGTGCTTCA
GCCGCTACCCCGACCACATGAAGCAGCACGACTTCTTCAAGTCCGCCATGCCCGAA
GGCTACGTCCAGGAGCGCACCATCTTCTTCAAGGGCGACGGCAACTACAAGACCCG
CGCCGAGGTGAAGTTCGAGGGCGACACCCTGGTGAACCGCATCGAGCTGAAGGGCA
TCGACTTCAAGGAGGACGGCAACATCCTGGGGCACAAGCTGGAGTACAACTACAAC
AGCCACAACGTCTATATCATGGCCGACAAGCAGAAGAACGGCATCAAGGTGAACTT
CAAGATCCGCCACAACATCGAGGACGGCAGCGTGCAGCTCGCCGACCACTACCAGC
AGAACACCCCCATCGGCGACGGCCCCGTGCTGCTGCCCGACAACCACTACCTGAGC
ACCCAGTCCGCCCTGAGCAAAGACCCCAACGAGAAGCGCGATCACATGGTCCTGCT
GGAGTTCGTGACCGCCGCCGGGATCACTCTCGGCATGGACGAGCTGTACaagctt

pAc5-DmYPss-DmYP1-EGFP

tctagaATGAACCCCATGAGAGTGCTGAGCCTTCTGGCTTGCTTGGCGGTCGCCGCCTTG
GCCAAGCCCAATGGCCGTATGGACAACTCCGTCAACCAGGCATTGAAGCCGTCGCA
GTGGCTCTCCGGATCCCAGCTGGAGGCCATTCCCGCCCTCGACGAtTTCACCATTGAG
CGTCTGGAGAACATGAACCTGGAGCGTGGCGCCGAGCTGCTGCAGCAAGTATACGG
GCCCgagctcCACCTGTCGCAGATCCACCACAACGTTGAGCCCAACTATGTGCCCAGCG
GCATCCAGGTCTATGTGCCCAAGCCCAATGGTGACAAGACCGTTGCTCCCCTGAACG
AGATGATCCAGCGCCTGAAGCAGAAGCAGAACTTTGGTGAGGATGAGGTGACCATC
ATTGTGACCGGACTGCCCCAGACCAGCGAGACCGTGAAGAAGGCGACCAGGAAGCT
GGTTCAGGCTTACATGCAGCGCTACAATCTGCAGCAGCAGCGCCAGCACGGCAAGA
ACGGCAACCAGGACTACCAGGATCAGAGCAACGAACAGAGGAAGAACCAGAGGAC
CAGCAGCGAGGAGGACTACAGCGAGGAGGTTAAGAACGCCAAGACCCAAAGCGGC
GACATCATTGTGATCGATTTGGGCTCCAAGCTGAACACCTATGAGCGTTATGCCATG

CTCGACATTGAGAAGACCGGCGCCAAGATCGGCAAGTGGATCGTCCAGATGGTCAA
CGAGTTGGACATGCCCTTCGATACCATTCACCTGATTGGCCAGAATGTGGGTGCCCA
TGTTGCCGGTGCCGCTGCCCAGGAATTCACCCGTCTCACCGGACACAAGCTGCGCCG
TGTCACCGGTCTGGATCCCTCCAAGATCGTGGCCAAGAGCAAGAACACCCTGACCG
GTCTGGCTCGCGGTGATGCTGAATTCGTTGACGCCATCCACACCTCGGTCTACGGCA
TGGGCACCCCCATCCGCTCCGGTGATGTTGACTTCTATCCCAATGGACCTGCCGCCG
GTGTTCCCGGAGCCAGCAACGTGGTGGAGGCCGCCATGCGTGCCACCCGCTACTTCG
CCGAGTCCGTGCGTCCCGGAAACGAGAGGAGCTTCCCCGCCGTGCCAGCCAACTCC
CTGCAGCAGTACAAGCAGAACGATGGATTCGGCAAGCGTGCCTACATGGGCATCGA
TACCGCTCACGATCTCGAGGGTGACTACATTCTGCAGGTGAACCCCAAGTCTCCTTT
CGGCCGCAACGCACCCGCCCAGAAGCAGAGCAGCTACCACGGTGTCCACCAGGCGT
GGAACACCAACCAGGACAGCAAGGACTACCAGgagctcAGGCGCGCCGGAGGAGGCG
GCAGCGGTGGTGGAGGAAGCGGTGGAGGCGGGAGCATGGTGAGCAAGGGCGAGGA
GCTGTTCACCGGGGTGGTGCCCATCCTGGTCGAGCTGGACGGCGACGTAAACGGCC
ACAAGTTCAGCGTGTCCGGCGAGGGCGAGGGCGATGCCACCTACGGCAAGCTGACC
CTGAAGTTCATCTGCACCACCGGCAAGCTGCCCGTGCCCTGGCCCACCCTCGTGACC
ACCCTGACCTACGGCGTGCAGTGCTTCAGCCGCTACCCCGACCACATGAAGCAGCA
CGACTTCTTCAAGTCCGCCATGCCCGAAGGCTACGTCCAGGAGCGCACCATCTTCTT
CAAGGGCGACGGCAACTACAAGACCCGCGCCGAGGTGAAGTTCGAGGGCGACACC
CTGGTGAACCGCATCGAGCTGAAGGGCATCGACTTCAAGGAGGACGGCAACATCCT
GGGGCACAAGCTGGAGTACAACTACAACAGCCACAACGTCTATATCATGGCCGACA
AGCAGAAGAACGGCATCAAGGTGAACTTCAAGATCCGCCACAACATCGAGGACGGC
AGCGTGCAGCTCGCCGACCACTACCAGCAGAACACCCCCATCGGCGACGGCCCCGT
GCTGCTGCCCGACAACCACTACCTGAGCACCCAGTCCGCCCTGAGCAAAGACCCCA
ACGAGAAGCGCGATCACATGGTCCTGCTGGAGTTCGTGACCGCCGCCGGGATCACT
CTCGGCATGGACGAGCTGTACaagctt

pAc5-DmYPss-P2-EGFP

tctagaATGAACCCCATGAGAGTGCTGAGCCTTCTGGCTTGCTTGGCGGTCGCCGCCTTG
GCCAAGCCCAATGGCCGTATGGACAACTCCGTCAACCAGGCATTGAAGCCGTCGCA
GTGGCTCTCCGGATCCCAGCTGGAGGCCATTCCCGCCCTCGACGAtTTCACCATTGAG
CGTCTGGAGAACATGAACCTGGAGCGTGGCGCCGAGCTGCTGCAGCAAGTATACGG
GCCCgagctcCACCTGTCGCAGATCCACCACAACGTTGAGCCCAACTATGTGCCCAGCG
GCATCCAGGTCTATGTGCCCAAGCCCAATGGTGACAAGACCGTTGCTCCCCTGAACG
AGATGATCCAGCGCCTGAAGCAGAAGCAGAACTTTGGTGAGGATGAGGTGACCATC
ATTGTGACCGGACTGCCCCAGACCAGCGAGACCGTGAAGAAGGCGACCAGGAAGCT
GGTTCAGGCTTACATGCAGCGCTACAATCTGCAGCAGCAGCGCCAGCACGGCAAGA
ACGGCAACCAGGACTACCAGGATCAGAGCAACGAACAGAGGAAGAACCAGAGGAC
CAGCAGCGAGGAGGACTACAGCGAGGAGGTTAAGAACgagctcAGGCGCGCCGGAGG
AGGCGGCAGCGGTGGTGGAGGAAGCGGTGGAGGCGGGAGCATGGTGAGCAAGGGC
GAGGAGCTGTTCACCGGGGTGGTGCCCATCCTGGTCGAGCTGGACGGCGACGTAAA

CGGCCACAAGTTCAGCGTGTCCGGCGAGGGCGAGGGCGATGCCACCTACGGCAAGC
TGACCCTGAAGTTCATCTGCACCACCGGCAAGCTGCCCGTGCCCTGGCCCACCCTCG
TGACCACCCTGACCTACGGCGTGCAGTGCTTCAGCCGCTACCCCGACCACATGAAGC
AGCACGACTTCTTCAAGTCCGCCATGCCCGAAGGCTACGTCCAGGAGCGCACCATCT
TCTTCAAGGGCGACGGCAACTACAAGACCCGCGCCGAGGTGAAGTTCGAGGGCGAC
ACCCTGGTGAACCGCATCGAGCTGAAGGGCATCGACTTCAAGGAGGACGGCAACAT
CCTGGGGCACAAGCTGGAGTACAACTACAACAGCCACAACGTCTATATCATGGCCG
ACAAGCAGAAGAACGGCATCAAGGTGAACTTCAAGATCCGCCACAACATCGAGGAC
GGCAGCGTGCAGCTCGCCGACCACTACCAGCAGAACACCCCCATCGGCGACGGCCC
CGTGCTGCTGCCCGACAACCACTACCTGAGCACCCAGTCCGCCCTGAGCAAAGACC
CCAACGAGAAGCGCGATCACATGGTCCTGCTGGAGTTCGTGACCGCCGCCGGGATC
ACTCTCGGCATGGACGAGCTGTACaagctt

pAc5-DmYPss-P3-EGFP

tctagaATGAACCCCATGAGAGTGCTGAGCCTTCTGGCTTGCTTGGCGGTCGCCGCCTTG
GCCAAGCCCAATGGCCGTATGGACAACTCCGTCAACCAGGCATTGAAGCCGTCGCA
GTGGCTCTCCGGATCCCAGCTGGAGGCCATTCCCGCCCTCGACGAtTTCACCATTGAG
CGTCTGGAGAACATGAACCTGGAGCGTGGCGCCGAGCTGCTGCAGCAAGTATACGG
GCCCgagctcGCCAAGACCCAAAGCGGCGACATCATTGTGATCGATTTGGGCTCCAAGC
TGAACACCTATGAGCGTTATGCCATGCTCGACATTGAGAAGACCGGCGCCAAGATC
GGCAAGTGGATCGTCCAGATGGTCAACGAGTTGGACATGCCCTTCGATACCATTCAC
CTGATTGGCCAGAATGTGGGTGCCCATGTTGCCGGTGCCGCTGCCCAGGAATTCACC
CGTCTCACCGGACACAAGCTGCGCCGTGTCACCGGTCTGGATCCCTCCAAGATCGTG
GCCAAGAGCAAGAACACCCTGACCGGTCTGGCTCGCGGTGATGCTGAATTCGTTGA
CGCCATCCACACCTCGGTCTACGGCATGGGCgagctcAGGCGCGCCGGAGGAGGCGGC
AGCGGTGGTGGAGGAAGCGGTGGAGGCGGGAGCATGGTGAGCAAGGGCGAGGAGC
TGTTCACCGGGGTGGTGCCCATCCTGGTCGAGCTGGACGGCGACGTAAACGGCCAC
AAGTTCAGCGTGTCCGGCGAGGGCGAGGGCGATGCCACCTACGGCAAGCTGACCCT
GAAGTTCATCTGCACCACCGGCAAGCTGCCCGTGCCCTGGCCCACCCTCGTGACCAC
CCTGACCTACGGCGTGCAGTGCTTCAGCCGCTACCCCGACCACATGAAGCAGCACG
ACTTCTTCAAGTCCGCCATGCCCGAAGGCTACGTCCAGGAGCGCACCATCTTCTTCA
AGGGCGACGGCAACTACAAGACCCGCGCCGAGGTGAAGTTCGAGGGCGACACCCTG
GTGAACCGCATCGAGCTGAAGGGCATCGACTTCAAGGAGGACGGCAACATCCTGGG
GCACAAGCTGGAGTACAACTACAACAGCCACAACGTCTATATCATGGCCGACAAGC
AGAAGAACGGCATCAAGGTGAACTTCAAGATCCGCCACAACATCGAGGACGGCAGC
GTGCAGCTCGCCGACCACTACCAGCAGAACACCCCCATCGGCGACGGCCCCGTGCT
GCTGCCCGACAACCACTACCTGAGCACCCAGTCCGCCCTGAGCAAAGACCCCAACG
AGAAGCGCGATCACATGGTCCTGCTGGAGTTCGTGACCGCCGCCGGGATCACTCTCG
GCATGGACGAGCTGTACaagctt

pAc5-DmYPss-P4-EGFP

tctagaATGAACCCCATGAGAGTGCTGAGCCTTCTGGCTTGCTTGGCGGTCGCCGCCTTG
GCCAAGCCCAATGGCCGTATGGACAACTCCGTCAACCAGGCATTGAAGCCGTCGCA
GTGGCTCTCCGGATCCCAGCTGGAGGCCATTCCCGCCCTCGACGAtTTCACCATTGAG
CGTCTGGAGAACATGAACCTGGAGCGTGGCGCCGAGCTGCTGCAGCAAGTATACGG
GCCCgagctcACCCCCATCCGCTCCGGTGATGTTGACTTCTATCCCAATGGACCTGCCG
CCGGTGTTCCCGGAGCCAGCAACGTGGTGGAGGCCGCCATGCGTGCCACCCGCTAC
TTCGCCGAGTCCGTGCGTCCCGGAAACGAGAGGAGCTTCCCCGCCGTGCCAGCCAA
CTCCCTGCAGCAGTACAAGCAGAACGATGGATTCGGCAAGCGTGCCTACATGGGCA
TCGATACCGCTCACGATCTCGAGGGTGACTACATTCTGCAGGTGAACCCCAAGTCTC
CTTTCGGCCGCAACGCACCCGCCCAGAAGCAGAGCAGCTACCACGGTGTCCACCAG
GCGTGGAACACCAACCAGGACAGCAAGGACTACCAGgagctcAGGCGCGCCGGAGGA
GGCGGCAGCGGTGGTGGAGGAAGCGGTGGAGGCGGGAGCATGGTGAGCAAGGGCG
AGGAGCTGTTCACCGGGGTGGTGCCCATCCTGGTCGAGCTGGACGGCGACGTAAAC
GGCCACAAGTTCAGCGTGTCCGGCGAGGGCGAGGGCGATGCCACCTACGGCAAGCT
GACCCTGAAGTTCATCTGCACCACCGGCAAGCTGCCCGTGCCCTGGCCCACCCTCGT
GACCACCCTGACCTACGGCGTGCAGTGCTTCAGCCGCTACCCCGACCACATGAAGC
AGCACGACTTCTTCAAGTCCGCCATGCCCGAAGGCTACGTCCAGGAGCGCACCATCT
TCTTCAAGGGCGACGGCAACTACAAGACCCGCGCCGAGGTGAAGTTCGAGGGCGAC
ACCCTGGTGAACCGCATCGAGCTGAAGGGCATCGACTTCAAGGAGGACGGCAACAT
CCTGGGGCACAAGCTGGAGTACAACTACAACAGCCACAACGTCTATATCATGGCCG
ACAAGCAGAAGAACGGCATCAAGGTGAACTTCAAGATCCGCCACAACATCGAGGAC
GGCAGCGTGCAGCTCGCCGACCACTACCAGCAGAACACCCCCATCGGCGACGGCCC
CGTGCTGCTGCCCGACAACCACTACCTGAGCACCCAGTCCGCCCTGAGCAAAGACC
CCAACGAGAAGCGCGATCACATGGTCCTGCTGGAGTTCGTGACCGCCGCCGGGATC
ACTCTCGGCATGGACGAGCTGTACaagctt

pAc5-DmYPss-P5-EGFP

tctagaATGAACCCCATGAGAGTGCTGAGCCTTCTGGCTTGCTTGGCGGTCGCCGCCTTG
GCCAAGCCCAATGGCCGTATGGACAACTCCGTCAACCAGGCATTGAAGCCGTCGCA
GTGGCTCTCCGGATCCCAGCTGGAGGCCATTCCCGCCCTCGACGAtTTCACCATTGAG
CGTCTGGAGAACATGAACCTGGAGCGTGGCGCCGAGCTGCTGCAGCAAGTATACGG
GCCCgagctcCACCTGTCGCAGATCCACCACAACGTTGAGCCCAACTATGTGCCCAGCG
GCATCCAGGTCTATGTGCCCAAGCCCAATGGTGACAAGACCGTTGCTCCCCTGAACG
AGATGATCCAGCGCCTGAAGCAGAAGCAGAACTTTGGTGAGGATGAGGTGACCATC
ATTGTGACCGGACTGCCCCAGACCAGCGAGACCGTGAAGAAGGCGACCAGGAAGCT
GGTTCAGGCTTACATGCAGCGCTACAATCTGCAGCAGCAGCGCCAGCACGGCAAGA
ACGGCAACCAGGACTACCAGGATCAGAGCAACGAACAGAGGAAGAACCAGAGGAC
CAGCAGCGAGGAGGACTACAGCGAGGAGGTTAAGAACGCCAAGACCCAAAGCGGC
GACATCATTGTGATCGATTTGGGCTCCAAGCTGAACACCTATGAGCGTTATGCCATG
CTCGACATTGAGAAGACCGGCGCCAAGATCGGCAAGTGGATCGTCCAGATGGTCAA

CGAGTTGGACATGCCCTTCGATACCATTCACCTGATTGGCCAGAATGTGGGTGCCCA
TGTTGCCGGTGCCGCTGCCCAGGAATTCACCCGTCTCACCGGACACAAGCTGCGCCG
TGTCACCGGTCTGGATCCCTCCAAGATCGTGGCCAAGAGCAAGAACACCCTGACCG
GTCTGGCTCGCGGTGATGCTGAATTCGTTGACGCCATCCACACCTCGGTCTACGGCA
TGGGCgagctcAGGCGCGCCGGAGGAGGCGGCAGCGGTGGTGGAGGAAGCGGTGGAG
GCGGGAGCATGGTGAGCAAGGGCGAGGAGCTGTTCACCGGGGTGGTGCCCATCCTG
GTCGAGCTGGACGGCGACGTAAACGGCCACAAGTTCAGCGTGTCCGGCGAGGGCGA
GGGCGATGCCACCTACGGCAAGCTGACCCTGAAGTTCATCTGCACCACCGGCAAGC
TGCCCGTGCCCTGGCCCACCCTCGTGACCACCCTGACCTACGGCGTGCAGTGCTTCA
GCCGCTACCCCGACCACATGAAGCAGCACGACTTCTTCAAGTCCGCCATGCCCGAA
GGCTACGTCCAGGAGCGCACCATCTTCTTCAAGGGCGACGGCAACTACAAGACCCG
CGCCGAGGTGAAGTTCGAGGGCGACACCCTGGTGAACCGCATCGAGCTGAAGGGCA
TCGACTTCAAGGAGGACGGCAACATCCTGGGGCACAAGCTGGAGTACAACTACAAC
AGCCACAACGTCTATATCATGGCCGACAAGCAGAAGAACGGCATCAAGGTGAACTT
CAAGATCCGCCACAACATCGAGGACGGCAGCGTGCAGCTCGCCGACCACTACCAGC
AGAACACCCCCATCGGCGACGGCCCCGTGCTGCTGCCCGACAACCACTACCTGAGC
ACCCAGTCCGCCCTGAGCAAAGACCCCAACGAGAAGCGCGATCACATGGTCCTGCT
GGAGTTCGTGACCGCCGCCGGGATCACTCTCGGCATGGACGAGCTGTACaagctt

pAc5-DmYPss-P6-EGFP

tctagaATGAACCCCATGAGAGTGCTGAGCCTTCTGGCTTGCTTGGCGGTCGCCGCCTTG
GCCAAGCCCAATGGCCGTATGGACAACTCCGTCAACCAGGCATTGAAGCCGTCGCA
GTGGCTCTCCGGATCCCAGCTGGAGGCCATTCCCGCCCTCGACGAtTTCACCATTGAG
CGTCTGGAGAACATGAACCTGGAGCGTGGCGCCGAGCTGCTGCAGCAAGTATACGG
GCCCgagctcGCCAAGACCCAAAGCGGCGACATCATTGTGATCGATTTGGGCTCCAAGC
TGAACACCTATGAGCGTTATGCCATGCTCGACATTGAGAAGACCGGCGCCAAGATC
GGCAAGTGGATCGTCCAGATGGTCAACGAGTTGGACATGCCCTTCGATACCATTCAC
CTGATTGGCCAGAATGTGGGTGCCCATGTTGCCGGTGCCGCTGCCCAGGAATTCACC
CGTCTCACCGGACACAAGCTGCGCCGTGTCACCGGTCTGGATCCCTCCAAGATCGTG
GCCAAGAGCAAGAACACCCTGACCGGTCTGGCTCGCGGTGATGCTGAATTCGTTGA
CGCCATCCACACCTCGGTCTACGGCATGGGCACCCCCATCCGCTCCGGTGATGTTGA
CTTCTATCCCAATGGACCTGCCGCCGGTGTTCCCGGAGCCAGCAACGTGGTGGAGGC
CGCCATGCGTGCCACCCGCTACTTCGCCGAGTCCGTGCGTCCCGGAAACGAGAGGA
GCTTCCCCGCCGTGCCAGCCAACTCCCTGCAGCAGTACAAGCAGAACGATGGATTC
GGCAAGCGTGCCTACATGGGCATCGATACCGCTCACGATCTCGAGGGTGACTACATT
CTGCAGGTGAACCCCAAGTCTCCTTTCGGCCGCAACGCACCCGCCCAGAAGCAGAG
CAGCTACCACGGTGTCCACCAGGCGTGGAACACCAACCAGGACAGCAAGGACTACC
AGgagctcAGGCGCGCCGGAGGAGGCGGCAGCGGTGGTGGAGGAAGCGGTGGAGGCG
GGAGCATGGTGAGCAAGGGCGAGGAGCTGTTCACCGGGGTGGTGCCCATCCTGGTC
GAGCTGGACGGCGACGTAAACGGCCACAAGTTCAGCGTGTCCGGCGAGGGCGAGG
GCGATGCCACCTACGGCAAGCTGACCCTGAAGTTCATCTGCACCACCGGCAAGCTG

CCCGTGCCCTGGCCCACCCTCGTGACCACCCTGACCTACGGCGTGCAGTGCTTCAGC
CGCTACCCCGACCACATGAAGCAGCACGACTTCTTCAAGTCCGCCATGCCCGAAGG
CTACGTCCAGGAGCGCACCATCTTCTTCAAGGGCGACGGCAACTACAAGACCCGCG
CCGAGGTGAAGTTCGAGGGCGACACCCTGGTGAACCGCATCGAGCTGAAGGGCATC
GACTTCAAGGAGGACGGCAACATCCTGGGGCACAAGCTGGAGTACAACTACAACAG
CCACAACGTCTATATCATGGCCGACAAGCAGAAGAACGGCATCAAGGTGAACTTCA
AGATCCGCCACAACATCGAGGACGGCAGCGTGCAGCTCGCCGACCACTACCAGCAG
AACACCCCCATCGGCGACGGCCCCGTGCTGCTGCCCGACAACCACTACCTGAGCAC
CCAGTCCGCCCTGAGCAAAGACCCCAACGAGAAGCGCGATCACATGGTCCTGCTGG
AGTTCGTGACCGCCGCCGGGATCACTCTCGGCATGGACGAGCTGTACaagctt

pAc5-DmYPss-P2A-EGFP

tctagaATGAACCCCATGAGAGTGCTGAGCCTTCTGGCTTGCTTGGCGGTCGCCGCCTTG
GCCAAGCCCAATGGCCGTATGGACAACTCCGTCAACCAGGCATTGAAGCCGTCGCA
GTGGCTCTCCGGATCCCAGCTGGAGGCCATTCCCGCCCTCGACGAtTTCACCATTGAG
CGTCTGGAGAACATGAACCTGGAGCGTGGCGCCGAGCTGCTGCAGCAAGTATACGG
GCCCgagctcCACCTGTCGCAGATCCACCACAACGTTGAGCCCAACTATGTGCCCAGCG
GCATCCAGGTCTATGTGCCCAAGCCCAATGGTGACAAGACCGTTGCTCCCCTGAACG
AGATGATCCAGCGCCTagagctcAGGCGCGCCGGAGGAGGCGGCAGCGGTGGTGGAGG
AAGCGGTGGAGGCGGGAGCATGGTGAGCAAGGGCGAGGAGCTGTTCACCGGGGTG
GTGCCCATCCTGGTCGAGCTGGACGGCGACGTAAACGGCCACAAGTTCAGCGTGTC
CGGCGAGGGCGAGGGCGATGCCACCTACGGCAAGCTGACCCTGAAGTTCATCTGCA
CCACCGGCAAGCTGCCCGTGCCCTGGCCCACCCTCGTGACCACCCTGACCTACGGCG
TGCAGTGCTTCAGCCGCTACCCCGACCACATGAAGCAGCACGACTTCTTCAAGTCCG
CCATGCCCGAAGGCTACGTCCAGGAGCGCACCATCTTCTTCAAGGGCGACGGCAAC
TACAAGACCCGCGCCGAGGTGAAGTTCGAGGGCGACACCCTGGTGAACCGCATCGA
GCTGAAGGGCATCGACTTCAAGGAGGACGGCAACATCCTGGGGCACAAGCTGGAGT
ACAACTACAACAGCCACAACGTCTATATCATGGCCGACAAGCAGAAGAACGGCATC
AAGGTGAACTTCAAGATCCGCCACAACATCGAGGACGGCAGCGTGCAGCTCGCCGA
CCACTACCAGCAGAACACCCCCATCGGCGACGGCCCCGTGCTGCTGCCCGACAACC
ACTACCTGAGCACCCAGTCCGCCCTGAGCAAAGACCCCAACGAGAAGCGCGATCAC
ATGGTCCTGCTGGAGTTCGTGACCGCCGCCGGGATCACTCTCGGCATGGACGAGCTG
TACaagctt

pAc5-DmYPss-P2B-EGFP

tctagaATGAACCCCATGAGAGTGCTGAGCCTTCTGGCTTGCTTGGCGGTCGCCGCCTTG
GCCAAGCCCAATGGCCGTATGGACAACTCCGTCAACCAGGCATTGAAGCCGTCGCA
GTGGCTCTCCGGATCCCAGCTGGAGGCCATTCCCGCCCTCGACGAtTTCACCATTGAG
CGTCTGGAGAACATGAACCTGGAGCGTGGCGCCGAGCTGCTGCAGCAAGTATACGG
GCCCgagctcAAGCAGAAGCAGAACTTTGGTGAGGATGAGGTGACCATCATTGTGACC

GGACTGCCCCAGACCAGCGAGACCGTGAAGAAGGCGACCAGGAAGCTGGTTCAGG
CTTACATGCAGCGCTACAgggagctcAGGCGCGCCGGAGGAGGCGGCAGCGGTGGTGG
AGGAAGCGGTGGAGGCGGGAGCATGGTGAGCAAGGGCGAGGAGCTGTTCACCGGG
GTGGTGCCCATCCTGGTCGAGCTGGACGGCGACGTAAACGGCCACAAGTTCAGCGT
GTCCGGCGAGGGCGAGGGCGATGCCACCTACGGCAAGCTGACCCTGAAGTTCATCT
GCACCACCGGCAAGCTGCCCGTGCCCTGGCCCACCCTCGTGACCACCCTGACCTACG
GCGTGCAGTGCTTCAGCCGCTACCCCGACCACATGAAGCAGCACGACTTCTTCAAGT
CCGCCATGCCCGAAGGCTACGTCCAGGAGCGCACCATCTTCTTCAAGGGCGACGGC
AACTACAAGACCCGCGCCGAGGTGAAGTTCGAGGGCGACACCCTGGTGAACCGCAT
CGAGCTGAAGGGCATCGACTTCAAGGAGGACGGCAACATCCTGGGGCACAAGCTGG
AGTACAACTACAACAGCCACAACGTCTATATCATGGCCGACAAGCAGAAGAACGGC
ATCAAGGTGAACTTCAAGATCCGCCACAACATCGAGGACGGCAGCGTGCAGCTCGC
CGACCACTACCAGCAGAACACCCCCATCGGCGACGGCCCCGTGCTGCTGCCCGACA
ACCACTACCTGAGCACCCAGTCCGCCCTGAGCAAAGACCCCAACGAGAAGCGCGAT
CACATGGTCCTGCTGGAGTTCGTGACCGCCGCCGGGATCACTCTCGGCATGGACGAG
CTGTACaagctt

pAc5-DmYPss-P2C-EGFP

tctagaATGAACCCCATGAGAGTGCTGAGCCTTCTGGCTTGCTTGGCGGTCGCCGCCTTG
GCCAAGCCCAATGGCCGTATGGACAACTCCGTCAACCAGGCATTGAAGCCGTCGCA
GTGGCTCTCCGGATCCCAGCTGGAGGCCATTCCCGCCCTCGACGAtTTCACCATTGAG
CGTCTGGAGAACATGAACCTGGAGCGTGGCGCCGAGCTGCTGCAGCAAGTATACGG
GCCCgagctcAATCTGCAGCAGCAGCGCCAGCACGGCAAGAACGGCAACCAGGACTAC
CAGGATCAGAGCAACGAACAGAGGAAGAACCAGAGGACCAGCAGCGAGGAGGACT
ACAGCGAGGAGGTTAAGAACgagctcAGGCGCGCCGGAGGAGGCGGCAGCGGTGGTG
GAGGAAGCGGTGGAGGCGGGAGCATGGTGAGCAAGGGCGAGGAGCTGTTCACCGG
GGTGGTGCCCATCCTGGTCGAGCTGGACGGCGACGTAAACGGCCACAAGTTCAGCG
TGTCCGGCGAGGGCGAGGGCGATGCCACCTACGGCAAGCTGACCCTGAAGTTCATC
TGCACCACCGGCAAGCTGCCCGTGCCCTGGCCCACCCTCGTGACCACCCTGACCTAC
GGCGTGCAGTGCTTCAGCCGCTACCCCGACCACATGAAGCAGCACGACTTCTTCAAG
TCCGCCATGCCCGAAGGCTACGTCCAGGAGCGCACCATCTTCTTCAAGGGCGACGG
CAACTACAAGACCCGCGCCGAGGTGAAGTTCGAGGGCGACACCCTGGTGAACCGCA
TCGAGCTGAAGGGCATCGACTTCAAGGAGGACGGCAACATCCTGGGGCACAAGCTG
GAGTACAACTACAACAGCCACAACGTCTATATCATGGCCGACAAGCAGAAGAACGG
CATCAAGGTGAACTTCAAGATCCGCCACAACATCGAGGACGGCAGCGTGCAGCTCG
CCGACCACTACCAGCAGAACACCCCCATCGGCGACGGCCCCGTGCTGCTGCCCGAC
AACCACTACCTGAGCACCCAGTCCGCCCTGAGCAAAGACCCCAACGAGAAGCGCGA
TCACATGGTCCTGCTGGAGTTCGTGACCGCCGCCGGGATCACTCTCGGCATGGACGA
GCTGTACaagctt

Supplementary Note 2: DNA sequences for expression of fusion proteins P2C-EGFP, P2C-Cas9 and P2C-EGFP-Cas9 in the plasmid pET28a. Highlighted sequence in yellow is the 6xHis-Tag in the vector, dark red is for P2C, green is for EGFP and teal is for Cas9.

pET28a-P2C-EGFP

CATCATCATCATCATCACAGCAGCGGCCTGGTGCCGCGCGGCAGCCATATGGCTAGC
ATGACTGGTGGACAGCAAATGGGTCGCGGATCCAATCTGCAGCAGCAGCGCCAGCA
CGGCAAGAACGGCAACCAGGACTACCAGGATCAGAGCAACGAACAGAGGAAGAAC
CAGAGGACCAGCAGCGAGGAGGACTACAGCGAGGAGGTTAAGAACgagctcAGGCGC
GCCGGAGGAGGCGGCAGCGGTGGTGGAGGAAGCGGTGGAGGCGGGAGCATGGTGA
GCAAGGGCGAGGAGCTGTTCACCGGGGTGGTGCCCATCCTGGTCGAGCTGGACGGC
GACGTAAACGGCCACAAGTTCAGCGTGTCCGGCGAGGGCGAGGGCGATGCCACCTA
CGGCAAGCTGACCCTGAAGTTCATCTGCACCACCGGCAAGCTGCCCGTGCCCTGGCC
CACCCTCGTGACCACCCTGACCTACGGCGTGCAGTGCTTCAGCCGCTACCCCGACCA
CATGAAGCAGCACGACTTCTTCAAGTCCGCCATGCCCGAAGGCTACGTCCAGGAGC
GCACCATCTTCTTCAAGGGCGACGGCAACTACAAGACCCGCGCCGAGGTGAAGTTC
GAGGGCGACACCCTGGTGAACCGCATCGAGCTGAAGGGCATCGACTTCAAGGAGGA
CGGCAACATCCTGGGGCACAAGCTGGAGTACAACTACAACAGCCACAACGTCTATA
TCATGGCCGACAAGCAGAAGAACGGCATCAAGGTGAACTTCAAGATCCGCCACAAC
ATCGAGGACGGCAGCGTGCAGCTCGCCGACCACTACCAGCAGAACACCCCCATCGG
CGACGGCCCCGTGCTGCTGCCCGACAACCACTACCTGAGCACCCAGTCCGCCCTGAG
CAAAGACCCCAACGAGAAGCGCGATCACATGGTCCTGCTGGAGTTCGTGACCGCCG
CCGGGATCACTCTCGGCATGGACGAGCTGTACggattc

pET28a-P2C-Cas9

CATCATCATCATCATCACAGCAGCGGCCTGGTGCCGCGCGGCAGCCATATGGCTAGC
ATGACTGGTGGACAGCAAATGGGTCGCGGATCCAATCTGCAGCAGCAGCGCCAGCA
CGGCAAGAACGGCAACCAGGACTACCAGGATCAGAGCAACGAACAGAGGAAGAAC
CAGAGGACCAGCAGCGAGGAGGACTACAGCGAGGAGGTTAAGAACgagctcCGTCGA
CAAGCTTGCGGCCGCATGGACAAGAAGTACAGCATCGGCCTGGACATCGGTACCAA
CAGCGTGGGCTGGGCCGTGATCACCGACGAGTACAAGGTGCCCAGCAAGAAGTTCA
AGGTGCTGGGCAACACCGACCGCCACAGCATCAAGAAGAACCTGATCGGCGCCCTG
CTGTTCGACAGCGGCGAGACCGCCGAGGCCACCCGCCTGAAGCGCACCGCCCGCCG
CCGCTACACCCGCCGCAAGAACCGCATCTGCTACCTGCAGGAGATCTTCAGCAACG
AGATGGCCAAGGTGGACGACAGCTTCTTCCACCGCCTGGAGGAGAGCTTCCTGGTG
GAGGAGGACAAGAAGCACGAGCGCCACCCCATCTTCGGCAACATCGTGGACGAGGT
GGCCTACCACGAGAAGTACCCCACCATCTACCACCTGCGCAAGAAGCTGGTGGACA
GCACCGACAAGGCCGACCTGCGCCTGATCTACCTGGCCCTGGCCCACATGATCAAGT
TCCGCGGCCACTTCCTGATCGAGGGCGACCTGAACCCCGACAACAGCGACGTGGAC
AAGCTGTTCATCCAGCTGGTGCAGACCTACAACCAGCTGTTCGAGGAGAACCCCATC

AACGCCAGCGGCGTGGACGCCAAGGCCATCCTGAGCGCCCGCCTGAGCAAGAGCCG
CCGCCTGGAGAACCTGATCGCCCAGCTGCCCGGCGAGAAGAAGAACGGCCTGTTCG
GCAACCTGATCGCCCTGAGCCTGGGCCTGACCCCCAACTTCAAGAGCAACTTCGACC
TGGCCGAGGACGCCAAGCTGCAGCTGAGCAAGGACACCTACGACGACGACCTGGAC
AACCTGCTGGCCCAGATCGGCGACCAGTACGCCGACCTGTTCCTGGCCGCCAAGAA
CCTGAGCGACGCCATCCTGCTGAGCGACATCCTGCGCGTGAACACCGAGATCACCA
AGGCCCCCCTGAGCGCCAGCATGATCAAGCGCTACGACGAGCACCACCAGGACCTG
ACCCTGCTGAAGGCCCTGGTGCGCCAGCAGCTGCCCGAGAAGTACAAGGAGATCTT
CTTCGACCAGAGCAAGAACGGCTACGCCGGCTACATCGACGGCGGCGCCAGCCAGG
AGGAGTTCTACAAGTTCATCAAGCCCATCCTGGAGAAGATGGACGGCACCGAGGAG
CTGCTGGTGAAGCTGAACCGCGAGGACCTGCTGCGCAAGCAGCGCACCTTCGACAA
CGGCAGCATCCCCCACCAGATCCACCTGGGCGAGCTGCACGCCATCCTGCGCCGCC
AGGAGGACTTCTACCCCTTCCTGAAGGACAACCGCGAGAAGATCGAGAAGATCCTG
ACCTTCCGCATCCCCTACTACGTGGGCCCCCTGGCCCGCGGCAACAGCCGCTTCGCC
TGGATGACCCGCAAGAGCGAGGAGACCATCACCCCCTGGAACTTCGAGGAGGTGGT
GGACAAGGGCGCCAGCGCCCAGAGCTTCATCGAGCGCATGACCAACTTCGACAAGA
ACCTGCCCAACGAGAAGGTGCTGCCCAAGCACAGCCTGCTGTACGAGTACTTCACC
GTGTACAACGAGCTGACCAAGGTGAAGTACGTGACCGAGGGCATGCGCAAGCCCGC
CTTCCTGAGCGGCGAGCAGAAGAAGGCCATCGTGGACCTGCTGTTCAAGACCAACC
GCAAGGTGACCGTGAAGCAGCTGAAGGAGGACTACTTCAAGAAGATCGAGTGCTTC
GACAGCGTGGAGATCAGCGGCGTGGAGGACCGCTTCAACGCCAGCCTGGGCACCTA
CCACGACCTGCTGAAGATCATCAAGGACAAGGACTTCCTGGACAACGAGGAGAACG
AGGACATCCTGGAGGACATCGTGCTGACCCTGACCCTGTTCGAGGACCGCGAGATG
ATCGAGGAGCGCCTGAAGACCTACGCCCACCTGTTCGACGACAAGGTGATGAAGCA
GCTGAAGCGCCGCCGCTACACCGGCTGGGGCCGCCTGAGCCGCAAGCTTATCAACG
GCATCCGCGACAAGCAGAGCGGCAAGACCATCCTGGACTTCCTGAAGAGCGACGGC
TTCGCCAACCGCAACTTCATGCAGCTGATCCACGACGACAGCCTGACCTTCAAGGAG
GACATCCAGAAGGCCCAGGTGAGCGGCCAGGGCGACAGCCTGCACGAGCACATCGC
CAACCTGGCCGGCAGCCCCGCCATCAAGAAGGGCATCCTGCAGACCGTGAAGGTGG
TGGACGAGCTGGTGAAGGTGATGGGCCGCCACAAGCCCGAGAACATCGTGATCGAG
ATGGCCCGCGAGAACCAGACCACCCAGAAGGGCCAGAAGAACAGCCGCGAGCGCA
TGAAGCGCATCGAGGAGGGCATCAAGGAGCTGGGCAGCCAGATCCTGAAGGAGCA
CCCCGTGGAGAACACCCAGCTGCAGAACGAGAAGCTGTACCTGTACTACCTGCAGA
ACGGCCGCGACATGTACGTGGACCAGGAGCTGGACATCAACCGCCTGAGCGACTAC
GACGTGGACCACATCGTGCCCCAGAGCTTCCTGAAGGACGACAGCATCGACAACAA
GGTGCTGACCCGCAGCGACAAGAACCGCGGCAAGAGCGACAACGTGCCCAGCGAG
GAGGTGGTGAAGAAGATGAAGAACTACTGGCGCCAGCTGCTGAACGCCAAGCTGAT
CACCCAGCGCAAGTTCGACAACCTGACCAAGGCCGAGCGCGGCGGCCTGAGCGAGC
TGGACAAGGCCGGCTTCATCAAGCGCCAGCTGGTGGAGACCCGCCAGATCACCAAG
CACGTGGCCCAGATCCTGGACAGCCGCATGAACACCAAGTACGACGAGAACGACAA
GCTGATCCGCGAGGTGAAGGTGATCACCCTGAAGAGCAAGCTGGTGAGCGACTTCC
GCAAGGACTTCCAGTTCTACAAGGTGCGCGAGATCAACAACTACCACCACGCCCAC
GACGCCTACCTGAACGCCGTGGTGGGCACCGCCCTGATCAAGAAGTACCCCAAGCT

GGAGAGCGAGTTCGTGTACGGCGACTACAAGGTGTACGACGTGCGCAAGATGATCG
CCAAGAGCGAGCAGGAGATCGGCAAGGCCACCGCCAAGTACTTCTTCTACAGCAAC
ATCATGAACTTCTTCAAGACCGAGATCACCCTGGCCAACGGCGAGATCCGCAAGCG
CCCCCTGATCGAGACCAACGGCGAGACCGGCGAGATCGTGTGGGACAAGGGCCGCG
ACTTCGCCACCGTGCGCAAGGTGCTGAGCATGCCCCAGGTGAACATCGTGAAGAAG
ACCGAGGTGCAGACCGGCGGCTTCAGCAAGGAGAGCATCCTGCCCAAGCGCAACAG
CGACAAGCTGATCGCCCGCAAGAAGGACTGGGACCCCAAGAAGTACGGCGGCTTCG
ACAGCCCCACCGTGGCCTACAGCGTGCTGGTGGTGGCCAAGGTGGAGAAGGGCAAG
AGCAAGAAGCTGAAGAGCGTGAAGGAGCTGCTGGGCATCACCATCATGGAGCGCAG
CAGCTTCGAGAAGAACCCCATCGACTTCCTGGAGGCCAAGGGCTACAAGGAGGTGA
AGAAGGACCTGATCATCAAGCTGCCCAAGTACAGCCTGTTCGAGCTGGAGAACGGC
CGCAAGCGCATGCTGGCCAGCGCCGGCGAGCTGCAGAAGGGCAACGAGCTGGCCCT
GCCCAGCAAGTACGTGAACTTCCTGTACCTGGCCAGCCACTACGAGAAGCTGAAGG
GCAGCCCCGAGGACAACGAGCAGAAGCAGCTGTTCGTGGAGCAGCACAAGCACTAC
CTGGACGAGATCATCGAGCAGATCAGCGAGTTCAGCAAGCGCGTGATCCTGGCCGA
CGCCAACCTGGACAAGGTGCTGAGCGCCTACAACAAGCACCGCGACAAGCCCATCC
GCGAGCAGGCCGAGAACATCATCCACCTGTTCACCCTGACCAACCTGGGCGCCCCC
GCCGCCTTCAAGTACTTCGACACCACCATCGACCGCAAGCGCTACACCAGCACCAA
GGAGGTGCTGGACGCCACCCTGATCCACCAGAGCATCACCGGTCTGTACGAGACCC
GCATCGACCTGAGCCAGCTGGGCGGCGACGGCGGCTCCGGACCTCCAAAGAAAAAG
AGAAAAGTATACCCCTACGACGTGCCCGACTACGCCTGTTAA

pET28a-P2C-EGFP-Cas9

CATCATCATCATCATCACAGCAGCGGCCTGGTGCCGCGCGGCAGCCATATGGCTAGC
ATGACTGGTGGACAGCAAATGGGTCGCGGATCCAATCTGCAGCAGCAGCGCCAGCA
CGGCAAGAACGGCAACCAGGACTACCAGGATCAGAGCAACGAACAGAGGAAGAAC
CAGAGGACCAGCAGCGAGGAGGACTACAGCGAGGAGGTTAAGAACgagctcAGGCGC
GCCGGAGGAGGCGGCAGCGGTGGTGGAGGAAGCGGTGGAGGCGGGAGCATGGTGA
GCAAGGGCGAGGAGCTGTTCACCGGGGTGGTGCCCATCCTGGTCGAGCTGGACGGC
GACGTAAACGGCCACAAGTTCAGCGTGTCCGGCGAGGGCGAGGGCGATGCCACCTA
CGGCAAGCTGACCCTGAAGTTCATCTGCACCACCGGCAAGCTGCCCGTGCCCTGGCC
CACCCTCGTGACCACCCTGACCTACGGCGTGCAGTGCTTCAGCCGCTACCCCGACCA
CATGAAGCAGCACGACTTCTTCAAGTCCGCCATGCCCGAAGGCTACGTCCAGGAGC
GCACCATCTTCTTCAAGGGCGACGGCAACTACAAGACCCGCGCCGAGGTGAAGTTC
GAGGGCGACACCCTGGTGAACCGCATCGAGCTGAAGGGCATCGACTTCAAGGAGGA
CGGCAACATCCTGGGGCACAAGCTGGAGTACAACTACAACAGCCACAACGTCTATA
TCATGGCCGACAAGCAGAAGAACGGCATCAAGGTGAACTTCAAGATCCGCCACAAC
ATCGAGGACGGCAGCGTGCAGCTCGCCGACCACTACCAGCAGAACACCCCCATCGG
CGACGGCCCCGTGCTGCTGCCCGACAACCACTACCTGAGCACCCAGTCCGCCCTGAG
CAAAGACCCCAACGAGAAGCGCGATCACATGGTCCTGCTGGAGTTCGTGACCGCCG
CCGGGATCACTCTCGGCATGGACGAGCTGTACGGATCCGAATTCgagctcCGTCGACAA

GCTTGCGGCCGCgagctcCGTCGACAAGCTTGCGGCCGCATGGACAAGAAGTACAGCA
TCGGCCTGGACATCGGTACCAACAGCGTGGGCTGGGCCGTGATCACCGACGAGTAC
AAGGTGCCCAGCAAGAAGTTCAAGGTGCTGGGCAACACCGACCGCCACAGCATCAA
GAAGAACCTGATCGGCGCCCTGCTGTTCGACAGCGGCGAGACCGCCGAGGCCACCC
GCCTGAAGCGCACCGCCCGCCGCCGCTACACCCGCCGCAAGAACCGCATCTGCTAC
CTGCAGGAGATCTTCAGCAACGAGATGGCCAAGGTGGACGACAGCTTCTTCCACCG
CCTGGAGGAGAGCTTCCTGGTGGAGGAGGACAAGAAGCACGAGCGCCACCCCATCT
TCGGCAACATCGTGGACGAGGTGGCCTACCACGAGAAGTACCCCACCATCTACCAC
CTGCGCAAGAAGCTGGTGGACAGCACCGACAAGGCCGACCTGCGCCTGATCTACCT
GGCCCTGGCCCACATGATCAAGTTCCGCGGCCACTTCCTGATCGAGGGCGACCTGAA
CCCCGACAACAGCGACGTGGACAAGCTGTTCATCCAGCTGGTGCAGACCTACAACC
AGCTGTTCGAGGAGAACCCCATCAACGCCAGCGGCGTGGACGCCAAGGCCATCCTG
AGCGCCCGCCTGAGCAAGAGCCGCCGCCTGGAGAACCTGATCGCCCAGCTGCCCGG
CGAGAAGAAGAACGGCCTGTTCGGCAACCTGATCGCCCTGAGCCTGGGCCTGACCC
CCAACTTCAAGAGCAACTTCGACCTGGCCGAGGACGCCAAGCTGCAGCTGAGCAAG
GACACCTACGACGACGACCTGGACAACCTGCTGGCCCAGATCGGCGACCAGTACGC
CGACCTGTTCCTGGCCGCCAAGAACCTGAGCGACGCCATCCTGCTGAGCGACATCCT
GCGCGTGAACACCGAGATCACCAAGGCCCCCCTGAGCGCCAGCATGATCAAGCGCT
ACGACGAGCACCACCAGGACCTGACCCTGCTGAAGGCCCTGGTGCGCCAGCAGCTG
CCCGAGAAGTACAAGGAGATCTTCTTCGACCAGAGCAAGAACGGCTACGCCGGCTA
CATCGACGGCGGCGCCAGCCAGGAGGAGTTCTACAAGTTCATCAAGCCCATCCTGG
AGAAGATGGACGGCACCGAGGAGCTGCTGGTGAAGCTGAACCGCGAGGACCTGCTG
CGCAAGCAGCGCACCTTCGACAACGGCAGCATCCCCCACCAGATCCACCTGGGCGA
GCTGCACGCCATCCTGCGCCGCCAGGAGGACTTCTACCCCTTCCTGAAGGACAACCG
CGAGAAGATCGAGAAGATCCTGACCTTCCGCATCCCCTACTACGTGGGCCCCCTGGC
CCGCGGCAACAGCCGCTTCGCCTGGATGACCCGCAAGAGCGAGGAGACCATCACCC
CCTGGAACTTCGAGGAGGTGGTGGACAAGGGCGCCAGCGCCCAGAGCTTCATCGAG
CGCATGACCAACTTCGACAAGAACCTGCCCAACGAGAAGGTGCTGCCCAAGCACAG
CCTGCTGTACGAGTACTTCACCGTGTACAACGAGCTGACCAAGGTGAAGTACGTGAC
CGAGGGCATGCGCAAGCCCGCCTTCCTGAGCGGCGAGCAGAAGAAGGCCATCGTGG
ACCTGCTGTTCAAGACCAACCGCAAGGTGACCGTGAAGCAGCTGAAGGAGGACTAC
TTCAAGAAGATCGAGTGCTTCGACAGCGTGGAGATCAGCGGCGTGGAGGACCGCTT
CAACGCCAGCCTGGGCACCTACCACGACCTGCTGAAGATCATCAAGGACAAGGACT
TCCTGGACAACGAGGAGAACGAGGACATCCTGGAGGACATCGTGCTGACCCTGACC
CTGTTCGAGGACCGCGAGATGATCGAGGAGCGCCTGAAGACCTACGCCCACCTGTT
CGACGACAAGGTGATGAAGCAGCTGAAGCGCCGCCGCTACACCGGCTGGGGCCGCC
TGAGCCGCAAGCTTATCAACGGCATCCGCGACAAGCAGAGCGGCAAGACCATCCTG
GACTTCCTGAAGAGCGACGGCTTCGCCAACCGCAACTTCATGCAGCTGATCCACGAC
GACAGCCTGACCTTCAAGGAGGACATCCAGAAGGCCCAGGTGAGCGGCCAGGGCG
ACAGCCTGCACGAGCACATCGCCAACCTGGCCGGCAGCCCCGCCATCAAGAAGGGC
ATCCTGCAGACCGTGAAGGTGGTGGACGAGCTGGTGAAGGTGATGGGCCGCCACAA
GCCCGAGAACATCGTGATCGAGATGGCCCGCGAGAACCAGACCACCCAGAAGGGCC
AGAAGAACAGCCGCGAGCGCATGAAGCGCATCGAGGAGGGCATCAAGGAGCTGGG

CAGCCAGATCCTGAAGGAGCACCCCGTGGAGAACACCCAGCTGCAGAACGAGAAG
CTGTACCTGTACTACCTGCAGAACGGCCGCGACATGTACGTGGACCAGGAGCTGGA
CATCAACCGCCTGAGCGACTACGACGTGGACCACATCGTGCCCCAGAGCTTCCTGA
AGGACGACAGCATCGACAACAAGGTGCTGACCCGCAGCGACAAGAACCGCGGCAA
GAGCGACAACGTGCCCAGCGAGGAGGTGGTGAAGAAGATGAAGAACTACTGGCGC
CAGCTGCTGAACGCCAAGCTGATCACCCAGCGCAAGTTCGACAACCTGACCAAGGC
CGAGCGCGGCGGCCTGAGCGAGCTGGACAAGGCCGGCTTCATCAAGCGCCAGCTGG
TGGAGACCCGCCAGATCACCAAGCACGTGGCCCAGATCCTGGACAGCCGCATGAAC
ACCAAGTACGACGAGAACGACAAGCTGATCCGCGAGGTGAAGGTGATCACCCTGAA
GAGCAAGCTGGTGAGCGACTTCCGCAAGGACTTCCAGTTCTACAAGGTGCGCGAGA
TCAACAACTACCACCACGCCCACGACGCCTACCTGAACGCCGTGGTGGGCACCGCC
CTGATCAAGAAGTACCCCAAGCTGGAGAGCGAGTTCGTGTACGGCGACTACAAGGT
GTACGACGTGCGCAAGATGATCGCCAAGAGCGAGCAGGAGATCGGCAAGGCCACC
GCCAAGTACTTCTTCTACAGCAACATCATGAACTTCTTCAAGACCGAGATCACCCTG
GCCAACGGCGAGATCCGCAAGCGCCCCCTGATCGAGACCAACGGCGAGACCGGCGA
GATCGTGTGGGACAAGGGCCGCGACTTCGCCACCGTGCGCAAGGTGCTGAGCATGC
CCCAGGTGAACATCGTGAAGAAGACCGAGGTGCAGACCGGCGGCTTCAGCAAGGA
GAGCATCCTGCCCAAGCGCAACAGCGACAAGCTGATCGCCCGCAAGAAGGACTGGG
ACCCCAAGAAGTACGGCGGCTTCGACAGCCCCACCGTGGCCTACAGCGTGCTGGTG
GTGGCCAAGGTGGAGAAGGGCAAGAGCAAGAAGCTGAAGAGCGTGAAGGAGCTGC
TGGGCATCACCATCATGGAGCGCAGCAGCTTCGAGAAGAACCCCATCGACTTCCTG
GAGGCCAAGGGCTACAAGGAGGTGAAGAAGGACCTGATCATCAAGCTGCCCAAGTA
CAGCCTGTTCGAGCTGGAGAACGGCCGCAAGCGCATGCTGGCCAGCGCCGGCGAGC
TGCAGAAGGGCAACGAGCTGGCCCTGCCCAGCAAGTACGTGAACTTCCTGTACCTG
GCCAGCCACTACGAGAAGCTGAAGGGCAGCCCCGAGGACAACGAGCAGAAGCAGC
TGTTCGTGGAGCAGCACAAGCACTACCTGGACGAGATCATCGAGCAGATCAGCGAG
TTCAGCAAGCGCGTGATCCTGGCCGACGCCAACCTGGACAAGGTGCTGAGCGCCTA
CAACAAGCACCGCGACAAGCCCATCCGCGAGCAGGCCGAGAACATCATCCACCTGT
TCACCCTGACCAACCTGGGCGCCCCCGCCGCCTTCAAGTACTTCGACACCACCATCG
ACCGCAAGCGCTACACCAGCACCAAGGAGGTGCTGGACGCCACCCTGATCCACCAG
AGCATCACCGGTCTGTACGAGACCCGCATCGACCTGAGCCAGCTGGGCGGCGACGG
CGGCTCCGGACCTCCAAAGAAAAAGAGAAAAGTATACCCCTACGACGTGCCCGACT
ACGCCTGTTAA
